# Surface Plasmon Resonance-Based Immunosensor for Igm Detection with Gold Nanoparticles

**DOI:** 10.3390/mi12091092

**Published:** 2021-09-10

**Authors:** Nilay Bereli, Monireh Bakhshpour, Aykut Arif Topçu, Adil Denizli

**Affiliations:** 1 Chemistry Department, Hacettepe University, Beytepe, 06800 Ankara, Turkey; bereli@hacettepe.edu.tr (N.B.); monir.b1985@gmail.com (M.B.); 2 Medical Laboratory Program, Vocational School of Health Services, Aksaray University, 68100 Aksaray, Turkey; aykuttopcu@aksaray.edu.tr

**Keywords:** human immunoglobulin M, biosensor, immunosensor, surface plasmon resonance, gold nanoparticle

## Abstract

In this work, a surface plasmon resonance (SPR) based immunosensor was prepared by the immobilization of the amine-functionalized gold nanoparticles (N-AuNPs) on the sensing surface to sense immunoglobulin M (IgM) antibodies in the aqueous solution and artificial plasma. The characterization studies of SPR based immunosensor for IgM detection were performed with scanning electron microscope (SEM), contact angle measurements, and ellipsometry. Kinetic studies for the IgM immunosensor were carried out in the range of 1.0 to 200 ng/mL IgM concentrations in an aqueous solution. The total IgM analysis time including adsorption, desorption, and regeneration cycles was nearly 10 min for the prepared immunosensor. The limit of detection (LOD) and limit of quantification (LOQ) were found as 0.08 and 0.26 ng/mL, respectively. The reusability of the proposed immunosensor was tested with 6 consecutive adsorption-desorption, and regeneration cycles. Also, enzyme-linked immunosorbent assay (ELISA) method was utilized in the validation of the immunosensor.

## 1. Introduction

IgM is the largest (950 kDa) and the first produced antibody in human serum to combat or stop the infections with the higher avidity of the antigens because of having 10 free antigen-binding sites [1,2,3,4]. The monitoring of IgM level in biological fluids is helpful to diagnose bacterial or viral infections from Dengue virus to some of certain diseases; including, acute chronic hepatitis, rheumatoid arthritis; furthermore, IgM deficiency is an immune disorder related to serious infections [2,5,6,7,8]. From this point of view, the detection of IgM levels in the biological fluids is of great importance for clinical studies to assess certain diseases and protect the public’s health [2,6,7,8,9]. Many methods commonly are used for detection of IgM. ELISA depended on the colorimetric method by using the enzyme-labelled antibodies is a widely adopted sensitive approach for the detection of antibodies [2,10]; however, the long incubation time, the need for multi-washing step, and the requirement of the labelled molecules are the main disadvantages for practical use of ELISA in clinical trials.

To overcome these limitations, SPR based sensors measuring the change in the refractive index near the sensing surface with real-time and label-free monitoring have been attracted more attention of the scientists because of the higher sensing capability of the various molecules including, the clinical analyte of interest [11,12,13]. Moreover, applying some surface modifications like the formation of the tailor-made receptors or antigen/antibody immobilization on the sensing surface could enhance the selectivity performance of the SPR based sensors against the target molecules such as antibiotics [14], neurotransmitters [15], and biomarkers as well [16,17,18].

Recently, the implementation of noble metal-based nanoparticles (NPs) such as Au and silver (Ag) NPs on SPR biosensors could enhance the sensitivity of SPR sensors owing to their capability of increasing the SPR responses [19,20,21,22,23]. For instance, streptavidin and bovine serum albumin (BSA)-functionalized Au NPS were integrated on the SPR platform to detect carcinoembryonic antigen (CEA) in human plasma [19]. In other studies, the SPR based sensors were designed with the help of Au and Ag NPs to monitor urinary tract infection, and LOD limits of both sensing platforms were highly satisfactory in the aqueous solutions and urine mimic samples [24,25].

Herein, we aimed to design the SPR based immunosensor with the assistance of Au NPs for IgM detection in the aqueous solution and artificial plasma. For this purpose, the sensing surface of the SPR sensor was firstly modified with 1,8-octanedithiol, following that the amine-functionalized gold nanoparticles (N-Au NPS) were immobilized on the modified sensing surface via Au and SH interactions. Then, anti-human IgMs were anchored on the N-Au NPs by [1-ethyl-3-(3-dimethyl aminopropyl)carbodiimide (EDC)/N-hydroxysuccinimide (NHS)] coupling reaction. The kinetic binding analyzes of hIgM were carried out by SPR based immunosensor sensor in the aqueous solution and artificial plasma. The selectivity studies of the prepared sensor were tested against immunoglobulin G (IgG) and hemoglobin (Hb) as competitor molecules in the aqueous solution, and the feasibility of the immunosensor was investigated in the artificial plasma.

## 2. Experimental

### 2.1. Materials

1,8-octanedithiol [(HSCH_2_(CH_2_)_6_CH_2_SH)], N-Au NPs (15 nm diameter, amine functionalized, 765325), IgM, anti-human IgM (a-hIgM), IgG, Hb, EDC, NHS and artificial plasma were purchased from Sigma Aldrich corporation (St. Louis, MO, USA). The other reagents as analytical and organic grade was supplied from Merck A.G and during the whole experimental process, deionized water (DW) was used.

### 2.2. Preparation of SPR Based Immunosensor

In the first step, the gold surface of the SPR chip was washed with acidic piranha solution [sulfuric acid/hydrogen peroxide (3:1 *v/v*)] for nearly 3 min, then was rinsed with DW, and dried in a vacuum oven (200 mmHg, 40 °C). After that, the gold sensing surface was modified according to a previous study [26] by using an ethanol solution containing 1,8-octanedithiol (4.3 mM) for 12 h, and the excess of 1,8-octanedithiol on the sensing surface was removed with an ethanol solution and rinsed with DW.

The modified surface was treated with 5 µL of the N-Au NPs for 3 h to attach the N-Au NPs, and then, the unbounded N-Au NPs was removed from the modified surface by using DW.

**Figure 1:**
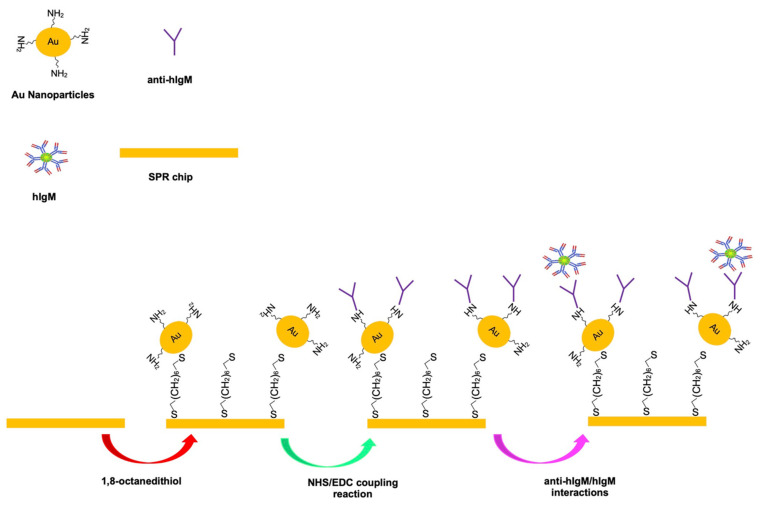
The schematic preparation of I-SPR sensor.

In the second step, a-hIgM was anchored onto the N-Au NPs by using the EDC-NHS coupling reaction via the amine groups of the Au NPs and the carboxyl groups of a-hIgM [27]. Before the coupling reaction occurred, 10 µL a-hIgM solution was dropped onto the surface, after that, the coupling reaction between the amine groups of Au NPs and the carboxyl groups of hIgM occurred in a solution containing 100 mM EDC and 25 mM NHS in DW for 15 min. Following that, the sensing surface was washed and rinsed with 100 mM phosphate buffer saline (PBS, pH 7.4) and DW, respectively. After the coupling reaction, the prepared immune SPR sensor was called as I-SPR sensor. The plain SPR (P-SPR) sensor was also prepared with the same methods that use for obtain of I-SPR sensor without using a-hIgM. The schematic preparation of I-SPR sensor was shown in Figure 1.

### 2.3. Characterization Studies

The sensing surface of the I-SPR sensor and P-SPR sensor were examined with SEM (GAIA3, Tescan, Brno, Czech Republic), contact angle (CA) and ellipsometer measurements. Before the SEM analysis, the I-SPR sensor surface was coated with gold following that, the images on the sensing surface were taken with different magnifications.

The surface wettability of the bare chip, I-SPR sensor, and P-SPR sensor were analyzed by CA measurements (KRUSS DSA100, Hamburg, Germany) with the sessile drop method and 5 different regions were chosen from the bare chip, the I-SPR, and the P-SPR sensors. The thickness of the 1,8-octanedithiol modified surface, the P-SPR and, the I-SPR sensors were investigated by ellipsometer measurements. The measurements were taken at 620 angles with 532 nm wavelength settings and the mean values were recorded by ellipsometry (Nanofilm EP3, Goetting, Hanover, Germany).

### 2.4. Real-Time Monitoring of IgM

SPR is an optical phenomenon and SPR based sensors measure the change in the refractive index (ΔR) close to the sensing surface (conductive metal and the electric medium) in real-time and label-free. In present study, SPRimager II (GWC Technologies, Azusa, CA, USA) was used (the operating wavelength 800 nm, the prism material SF 10 glass) in evaluating the kinetic analysis of IgM in the aqueous solution and artificial plasma. Before the experimental studies, the resonance angle was adjusted to the appropriate angle then, 1.0–200.0 ng/mL IgM solutions was sent to (150 mL/min) I-SPR at 25 °C.

During the experimental procedure, the sensor surface was firstly equilibrated with PBS buffer (100 mM, pH 7.4), then the IgM solution was passed through the sensor surface until the stable signals monitored. The increasing amounts of IgM results in enhancing the (ΔR) value. After that, the bounded IgM was removed from the sensor surface using 100 mM NaCI solution until the stable signals occurred. Finally, the sensor surface was cleaned with DW and the equilibrium buffer.

The artificial plasma was chosen to investigate the feasibility of the prepared immuno-sensor system. The plasma samples were firstly 10-folds diluted with PBS (7.4) buffer solution. Then, the plasma samples were prepared with the addition of different amounts of IgM (1.0, 5.0, and 10.0 ng/mL). The standard measurement of SPR system described above was used to determine the real-time monitoring of IgM detection from the artificial plasma.

### 2.5. Selectivity and Reusability Studies

The selectivity studies of the I-SPR sensor were performed against IgG and Hb molecules in the aqueous solution. The real-time detections of separate IgM, Hb, and IgG in IgM, Hb and IgG solutions and mixed (IgM + Hb + IgG) solutions were analyzed in order to determine the specificity of I-SPR sensor. The selectivity of I-SPR sensor was investigated at 50.0 ng/mL of each protein concentrations.

To examine the reusability of the I-SPR sensor, 5.0 ng/mL IgM solutions were prepared and interacted with the same sensing surface with consecutive six equilibration-binding and regeneration cycles. In each reusability cycle, the I-SPR sensor surface was firstly equilibrated with PBS buffer (100 mM, pH 7.4); afterwards, 5.0 ng/mL IgM solution was passed through (150 µL/min flow rate and at 25 °C) the I-SPR sensor until the stable signals monitored. Following that, the bounded hIgM were removed from the I-SPR using 100 mM NaCI solution and this procedure was carried out for six consecutive equilibration-binding and regeneration cycles. After the reusability studies, I-SPR was washed with DW and kept at 4 °C until used in further studies.

**Figure 2:**
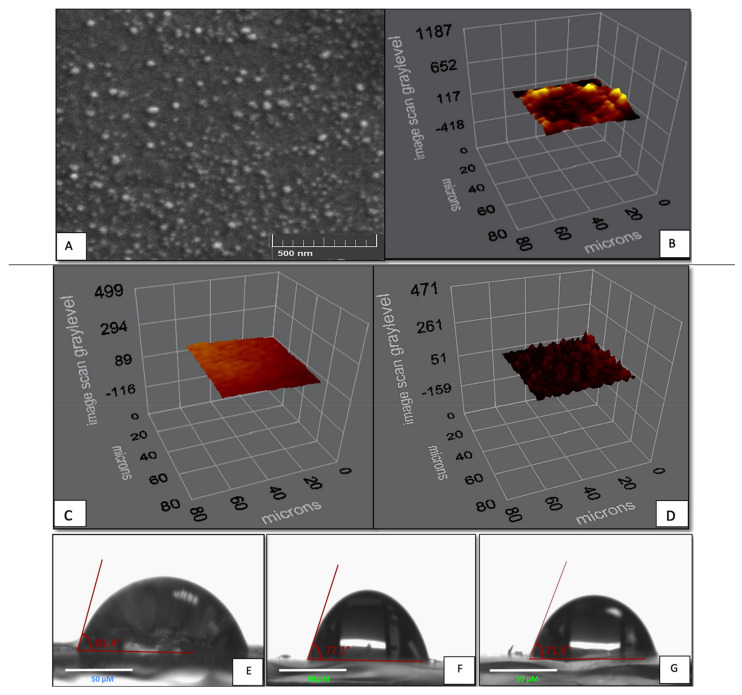
The SEM image (**A**) and the ellipsometer measurements of I-SPR sensor (**B**), 1,8-octanedithiol modified SPR chip (**C**), and P-SPR sensor (**D**), and CA measurements of the bare SPR chip (**E**), P-SPR sensor (**F**) and I-SPR sensor surfaces (**G**).

The immobilization of the N-Au NPs onto the I-SPR sensor surface was analyzed with the SEM and the image of the sensing surface was demonstrated in Figure 2. As shown in Figure 2A, the N-Au NPs was homogeneously distributed on the sensing surface without the aggregation. Hence, the N-Au NPs were successfully immobilized on the sensing surface by using the selected surface modification procedure. The ellipsometer measurements of the 1,8-octanedithiol modified SPR chip, P-SPR and the I-SPR sensors were shown in Figure 2B–D, respectively. The thicknesses of sensing surfaces were determined 10.9 ± 1.3 nm for the 1,8-octanedithiol modified SPR surface, 24.3 ± 1.4 nm for P-SPR sensor surface, and 30.2 ± 2.1 nm for I-SPR sensor surface. The immobilization of the N-Au NPs could improve the surface thickness of the P-SPR and I-SPR sensors and, these results showed that the immobilization N-Au NPs were successfully achieved on both sensor surfaces. Furthermore, the immobilization of a-hIgM on the I-SPR sensor could increase surface thickness of the I-SPR sensor compared to P-SPR sensor.

The CA measurements were performed to understand the wetting behavior of the bare SPR chip, P-SPR and I-SPR sensors. The measurement results were shown in Figure 2E–G, respectively. The surface wettability of the bare SPR chip and P-SPR sensor surface were calculated as 81.4 ± 1.9° and 77.1 ± 1.5° respectively, while the surface wettability of the I-SPR sensor surface was found 71.6 ± 1.5°. The attachment of N-Au-NPs on the P-SPR sensor could increase the surface hydrophilicity of the P-SPR sensor thanks to the hydrophilic amine groups of the Au-NPs. After the coupling of anti-IgM via the carboxyl domains of anti-IgM and the amine groups of the Au NPs [28] there was an enhancement in surface wettability of I-SPR sensor due to the hydrophilic groups of anti-IgM. The CA measurement results supported that the surface modifications were successfully achieved in each modification steps and, the successful incorporation of the hydrophilic groups enhanced the surface wettability.

## 3. Real-Time Monitoring of IgM Detection in Aqueous Solution and Artificial Plasma

The effect of pH on IgM detection using the I-SPR sensor was evaluated in the range of pH 4.0–8.0 buffer solutions as shown in presented Supplementary Materials Figure S1. To determine the optimum pH value for IgM detection in the aqueous solution, the sensing surface was equilibrated with the desired pH buffer solutions after that IgM solution (100 ng/mL) was interacted with I-SPR sensor. The results of the pH effect on IgM detection demonstrated that the increase of pH values caused an increase in the bounded amount of IgM on the sensing surface and the maximum %∆R value (9.95) was found at pH 7.4 phosphate buffer solution. But, over pH 7.4, the I-SPR sensor responses were sharply decreased because the specific binding sites of antigens and antibodies consisting of some amino acid derivates could be affected by changing the pH. So, over pH 7.4, the same charged groups of IgM and a-hIgM probably led to repulse each other and caused to decrease the bounded amount of IgM on the sensing surface resulting in decreased SPR signals. In this sense, the other experimental studies were carried out in pH 7.4 buffer solution in the aqueous solution and artificial plasma.

**Figure 3:**
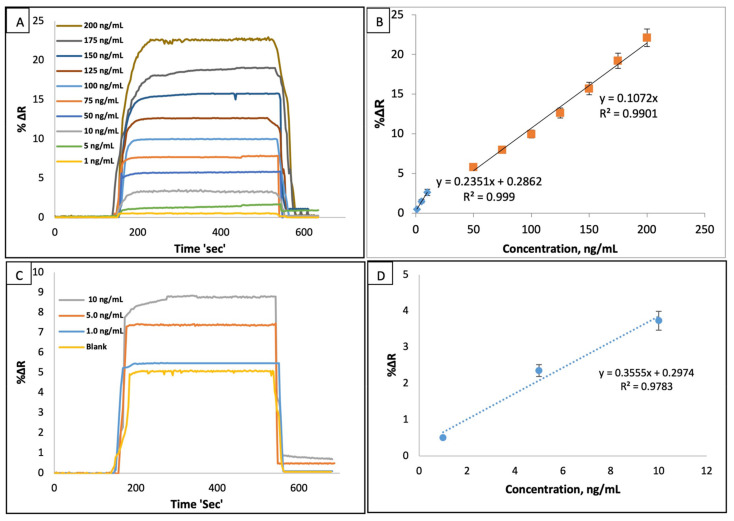
The responses and the linearity of the I-SPR sensor for IgM concentrations (**A**), (**B**) in the aqueous solution; (flow rate: 150 µL/min and T: 25 °C), and the responses and the linearity of the I-SPR sensor (**C**), (**D**) in the artificial plasma (flow rate: 150 µL/min, pH: 7.4 and T: 25 °C).

The SPR responses of the I-SPR sensor was examined in the range of 1.0–200 ng/mL IgM concentrations at 25 °C and, the concentrations of IgM versus the SPR signals (%∆R) were given in Figure 3A. The dynamic responses of I-SPR sensor were gradually increased by the increasing amounts of IgM solutions in the aqueous solution and the change of the IgM concentrations leading to a driving force caused to change in SPR signals. In Figure 3B, the correlation coefficient (R^2^) of the I-SPR sensor calculated as 0.99 showed that the developed immune-sensor had capable of IgM sensing in the aqueous solution. The response time for IgM adsorption, desorption and regeneration cycles was nearly about 10 min for this SPR based immunosensor system.

LOD and LOQ of the I-SPR sensor were calculated by using LOD = 3σ/s and LOQ = 10σ/s equations, respectively [29]. Herein, σ; is the standard deviation of the I-SPR sensor response and s; is the slope of the calibration curve. The standard deviation was also calculated using the blank samples for the minimum IgM concentration (1.0 ng/mL) (n = 10). The calculation results of LOD and the LOQ values were found as 0.08 ng/mL and 0.26 ng/mL, respectively in the aqueous solution.

The IgM level is of great importance for clinical studies; so, the several sensor platforms were fabricated for IgM sensing. From this point of view, our experimental results were compared with the several sensor platforms according to the sensing method, linear range, LOD and showed in Table 1.

The artificial plasma was selected as a natural source for examining the feasibility of the proposed sensor and for this purpose, the diluted (1/10) plasma samples spiked with different amounts of IgM (1.0, 5.0 and 10.0 ng/mL) solutions were applied to I-SPR. The recognition ability of the I-SPR sensor was highly favorable in the artificial plasma with an R^2^ (0.98) value (Figure 3D), and the increasing amounts of IgM concentrations caused to increase in the I-SPR responses (Figure 3C); hence, I-SPR has a potential for IgM sensing in clinical studies. Also, hIgM ELISA Kit from Elabscience Biotechnology Inc., Houston, United States (detection limit; 3.13–200 ng/mL and sensitivity; 1.88 ng/mL) was used in the validation study of the immuno sensor. The IgM concentration in the non-diluted plasma was found as 51.6 ng/mL and the same concentration of IgM was calculated as 47.3 ng/mL using ELISA. The accuracy result of I-SPR (91.7%) was highly satisfactory to be used in clinical studies.

The adsorption isotherm models play a key role in describing the binding process, in this work, the Langmuir, the Freundlich, and the Langmuir-Freundlich adsorption isotherm models were applied to show the binding of IgM on the sensing surface [29]. The Langmuir, Freundlich, and Langmuir-Freundlich models and equations were explained in the Supplementary Materials. As illustrated in Table S1, our experimental results were close to the Langmuir isotherm model with a high correlation coefficient value R^2^ (0.99), and the sensing surface had a monolayer with the equal binding sites.

### Selectivity and Reusability Studies

**Figure 4:**
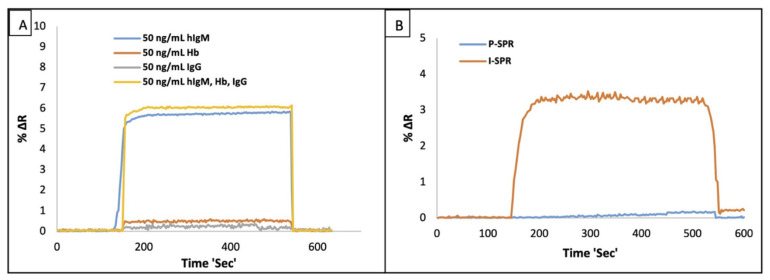
The selectivity of the I-SPR sensor (**A**) towards IgG and Hb molecules (concentration of the competitor molecules 50 ng/mL, pH: 7.4., flow rate: 150 µL/min and T: 25 °C) and the comparison results of I-SPR and P-SPR (**B**) in the aqueous solution (IgM concentration: 10 ng/mL, pH: 7.4., flow rate: 150 µL/min and T: 25 °C).

In this work, we tried to develop the SPR based immunosensor by the anchoring of a-IgM on N-Au NPs for IgM detection in real-time and without labeled molecules in the aqueous solution and artificial plasma. To test the selectivity of the I-SPR sensor, IgG and Hb were chosen as competitor molecules because IgG is the same family with IgM and Hb was selected owing to present in plasma. For this aim, 50.0 ng/mL of IgM, Hb, and IgG solutions, and their mixtures were used to test the selectivity of the I-SPR sensor, and the selectivity results were shown in Figure 4A. As seen in Figure 4A, the responses of the I-SPR sensor against IgM were more notable than the competitor molecules; so, in the light of the experimental results, I-SPR sensor was capable of selectively recognize IgM in the aqueous solution.

The IgM sensing performances of the I-SPR sensor and the P-SPR sensor were given in Figure 4B. The recognition performance of the I-SPR sensor was quite higher than the P-SPR because the immobilization of N-AuNPs and the anchoring of a-hIgM onto the N-AuNPs could enhance the dynamic responses of the I-SPR.

**Figure 5:**
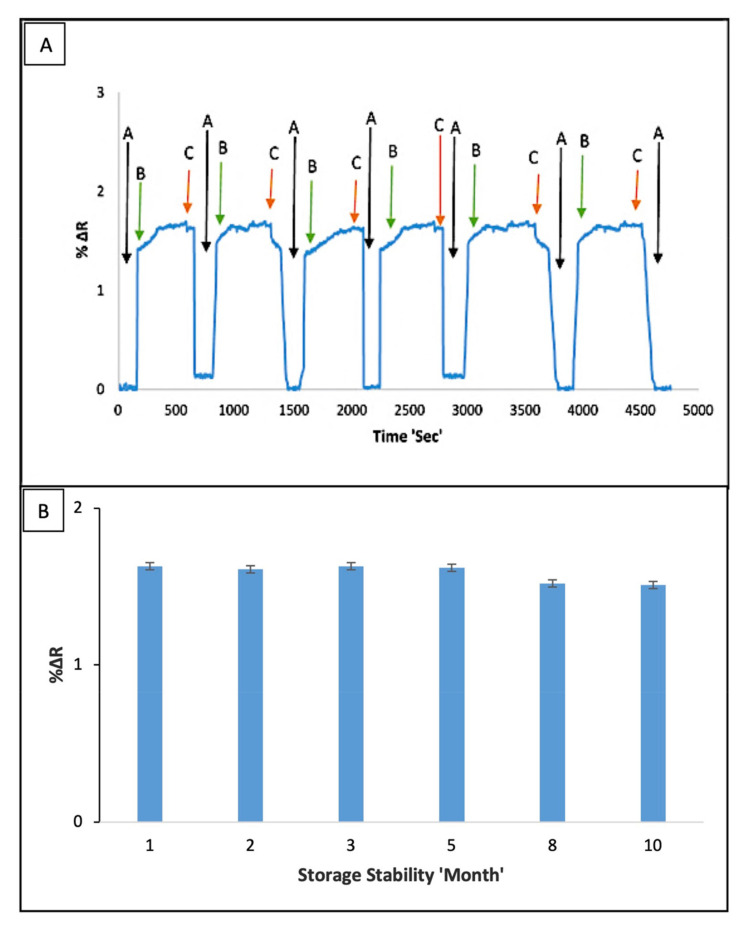
(**A**) The reusability of the I-SPR sensor (“A”: equilibration, “B”: binding, “C”: desorption), (**B**) The storage stability results of the I-SPR sensor in aqueous solution within 1–10 month periods (hIgM concentration: 5 ng/mL, pH: 7.4., flow rate: 150 µL/min and T: 25 °C).

A perfect sensor possesses well sensitivity and reusability. From this point of view, the reusability of studies was examined by detecting IgM at a fixed concentration by using same the I-SPR. For this aim, the same sensor chip was chosen and tested with six consecutive binding-rebinding, and regeneration cycles. The reusability results were given in Figure 5A. The result displayed the positive reusability of the I-SPR and the nearly same SPR responses were detected in the whole binding process. The I-SPR sensor maintained its stability during the 6 equilibration-binding and regeneration cycles without the notable changes in signal responses. Also, after 6 repeated cycles, the binding capacity of I-SPR sensor was not decreased.

In addition, the storage stability of the I-SPR sensor was tested on the different periods (1–10 month) and before the storage stability tests, the I-SPR sensor was kept nearly for 30 min at room temperature to reach 25 °C. Following that, the I-SPR was equilibrated with PBS buffer (100 mM, pH 7.4) and 5.0 ng/mL IgM solution was passed through (150 µL/min) the I-SPR sensor at 25 °C. After the stable signals occurred, 100 mM NaCl solution was used in removing the bounded hIgM from the I-SPR sensor, and the I-SPR sensor was washed with DW and kept at 4 °C until used. This procedure abovementioned was applied in each step of the storage stability test (1–10 month) and the storage stability of the I-SPR was obtained as 94.23% (Figure 5B); so, the developed immunosensor had capable of maintaining its stability over a long time.

## 4. Conclusions

ELISA is a well-established and highly selective method used in clinical studies depended on the antigen-antibody interactions; however, the requirement of the labelled molecule, the cost, and the long incubation time are the major drawbacks of ELISA.

SPR based sensors measure a change in the refractive index at the sensing surface depending on analyte concentrations and this optical phenomenon allows to investigate of the binding kinetics of the molecules [37]. Before the design of a SPR sensor, the receptor is immobilized on a sensing surface and after that, the kinetic analysis of the target molecule is investigated in real time and without a need of labelled molecule.

In this work, we developed a SPR based immunosensor for IgM sensing in the aqueous solution and artificial plasma. For this purpose, the AuNPs were used as a signal enhancer and a-hIgM carrier. Before the design of the I-SPR sensor, the AuNPs were firstly anchored onto the modified surface. Following that, the a-hIgM antibodies were immobilized onto the N-AuNPs and after the characterization studies, the kinetic analyses of IgM were examined in the aqueous solution and artificial plasma.

The surface characterization results showed that the immobilization of the N-AuNPs was successfully achieved with homogeneously distributed on the sensing surface, and the successful surface modifications led to differences in the surface morphology of the sensing surface. The immobilization of N-AuNPs could increase the surface thickness and hydrophilicity of I-SPR compared to the modified SPR sensor chip, the P-SPR and the bare SPR sensor chip. The I-SPR sensor responses were found to be linear with a high R^2^ (0.99) value, and the binding of IgM fitted the Langmuir adsorption isotherm model with a monolayer adsorption surface in the aqueous solution. The immobilization of N-AuNPs onto the I-SPR sensor could enhance the signal amplification of the I-SPR and led to improving the LOD (0.08 ng/mL) and LOQ (0.26 ng/mL) values compared to the other sensing platforms (Table 1). The feasibility results of the I-SPR sensor in the artificial plasma were highly predictive with a high R^2^ (0.98); moreover, the accuracy result of the prepared sensor and ELISA was found over 90%.

In the light of the experimental findings, the proposed optical immunosensor platform could be potentially used in IgM detection among the other sensing platforms thanks to the short analysis time and the low LOD, without the need for the labelled molecule.

## Figures and Tables

**Table 1:** The comparison results of the other immunosensing platforms by using anti-hIgM antibodies for IgM detection according to sensing methods, linear ranges and the limit of detection (LOD) values.

Sensing Method	Linear Range	LOD	Reference
Optical fibre long period grafting biosensor	15.6 µg/mL–1.0 mg/mL	15 pg/mm^2^	[2]
Electrochemical	0–0.5 mg/mL	0.013 mg/mL	[3]
Surface-enhanced fluorescence	10 ng/mL–100 µg/mL	nearly 5 ng/mL	[30]
Giant magnetoresistance biosensor	50–2000 ng/mL	50 ng/mL	[31]
Lateral flow assay	0.3–1.2 mg/mL	no data	[32]
Electrochemical optical fibre biosensor	1–200 IU/mL	0.6–0.22 IU/mL	[33]
Optical fibre long period grating sensor	19 nM–0.3 µM	0.02 ng/mm^2^	[34]
DNA-assisted nanopore sensing	0.05–10 µg/mL	50 ng/mL	[35]
Magnetic based ELISA	0–100 µg/mL	0.8–40 ng/mL	[36]
SPR based immunosensor	1.0–200 ng/mL	0.08 ng/mL	in this work

## Supplementary Materials

The following are available online at www.mdpi.com/2072-666X/12/9/1092/s1, Figure S1: The effects of pH on the detection of IgM using I-SPR sensor (flow rate: 150 µL/min, 100 ng/mL IgM solution and T: 25 °C), Table S1: The results of the Langmuir, the Freundlich and the Langmuir-Freundlich adsorption isotherm models.

## References

[1] Fellah J.S., Wiles M.V., Charlemagne J., Schwager J. (1992). Evolution of vertebrate IgM: Complete amino acid sequence of the constant region of Ambystoma mexicanum mu chain deduced from cDNA sequence. Eur. J. Immunol..

[2] Liu L.L., Marques L., Correia R., Morgan S.P., Lee S.W., Tighe P., Fairclough L., Korposh S. (2018). Highly sensitive label-free antibody detection using a long period fibre grating sensor. Sens. Actuators B Chem..

[3] Ortega G.A., Zuaznabar-Gardona J.C., Reguer E. (2018). Electrochemical immunoassay for the detection of IgM antibodies using polydopamine particles loaded with PbS quantum dots as labels. Biosens. Bioelectron..

[4] Bakhshpour M., Topçu A.A., Bereli N., Alkan H., Denizli A. (2020). Poly(Hydroxyethyl Methacrylate) immunoaffinity cryogel column for the purification of human immunoglobulin M. Gels.

[5] Lima J.R.C., Rouquayrol M.Z., Callado M.R.M., Guedes M.I.F., Pessoa C. (2012). Interpretation of the presence of IgM and IgG antibodies in a rapid test for dengue: Analysis of dengue antibody prevalence in Fortaleza City in the 20th year of the epidemic. Rev. Soc. Bras. Med. Trop..

[6] Büyüktiryaki S., Yılmaz F., Say R., Ersöz A. (2019). Proteinous polymeric shell decorated nanocrystals for the recognition of immunoglobulin M. J. Fluoresc..

[7] Ehrenstein M.R., Notley C.A. (2010). The importance of natural IgM: Scavenger, protector and regulator. Nat. Rev. Immunol..

[8] Diltemiz S.E., Hür D., Keçili R., Ersöz A., Say R. (2013). New synthesis method for 4-MAPBA monomer and using for the recognition of IgM and mannose with MIP-based QCM sensors. Analyst.

[9] Chen P.J., Wang J., Hwang L., Yang Y., Hsieh C., Kau J. (1992). Transient immunoglobulin M antibody response to hepatitis C virus capsid antigen in posttransfusion hepatitis C: Putative serological marker for acute viral infection. Proc. Natl. Acad. Sci. USA.

[10] Hermanson G.T. (2013). Introduction to Bioconjugation, Bioconjugate Techniques.

[11] Masson J.F. (2017). Surface Plasmon Resonance Clinical Biosensors for Medical Diagnostics. ACS Sens..

[12] Sankiewicz A., Romanowicz L., Pyc M., Hermanowicz A., Gorodkiewicz E. (2018). SPR imaging biosensor for the quantification of fibronectin concentration in blood samples. J. Pharm. Biomed. Anal..

[13] Mariani S., Minunni M. (2014). Surface plasmon resonance applications in clinical analysis. Anal. Bioanal. Chem..

[14] Zhang L., Zhu C., Chen C., Zhu S., Zhou J., Wang M., Shang P. (2018). Determination of kanamycin using a molecularly imprinted SPR sensor. Food Chem..

[15] Pathak A., Gupta B.D. (2019). Ultra-selective fiber optic SPR platform for the sensing of dopamine in synthetic cerebrospinal fluid incorporating permselective nafion membrane and surface imprinted MWCNTs-Ppy matrix. Biosens. Bioelectron..

[16] Palladino P., Minnuni M., Scarano S. (2018). Cardiac Troponin T capture and detection in real-time via epitope-imprinted polymer and optical biosensing. Biosens. Bioelectron..

[17] Wang W., Mai Z., Chen Y., Wang J., Li L., Su Q., Li X., Hong X. (2017). A label-free fiber optic SPR biosensor for specific detection of C-reactive protein. Sci. Rep..

[18] Narayan T., Kumar S., Kumar S., Augustine S., Yadav B.K., Malhatra B.D. (2019). Protein functionalized I monolayer based biosensor for colon cancer detection. Talanta.

[19] Špringer T., Homola J. (2012). Biofunctionalized gold nanoparticles for SPR-biosensor-based detection of CEA in blood plasma. Anal Bional. Chem..

[20] Lao J.J., Han L.Z., Wu Z., Zhnag X.J., Huang Y.Y., Tang Y., Guo T. (2019). Gold nanoparticle functionalized surface plasmon resonance optical fiber biosensor: In situ detection of thrombin with 1 nM detection limit. J. Lightwave Technol..

[21] Shalabney A., Abdulhalim I. (2011). Sensitivity-enhancement methods for surface plasmon sensors. Laser Photonics Rev..

[22] Matsu J., Akamatsu K., Hara N., Miyoshi D., Nawafuna H., Tamaki K., Sugimoto N. (2005). SPR sensor chip for detection of small molecules using molecularly imprinted polymer with embedded gold nanoparticles. Anal. Chem..

[23] Cai W., Hofmesiter H., Rainer T., Chen W. (2001). Optical properties of Ag and Au nanoparticles dispersed within the pores of monolithic mesoporous silica. J. Nanopart. Res..

[24] Diken Gür S., Bakhshpour M., Denizli A. (2019). Selective detection of Escherichia coli caused UTIs with surface imprinted plasmonic nanoscale sensor. Mater. Sci. Eng. C.

[25] Özgür E., Topçu A.A., Yılmaz E., Denizli A. (2020). Surface plasmon resonance based biomimetic sensor for urinary tract infections. Talanta.

[26] Liu F., Huang L., Duan X., Li Y., Hu J., Li B., Lu J. (2018). A facile method to prepare noble metal nanoparticles modified Self-Assembly (SAM) electrode. J. Exp. Nanosci..

[27] Park J.H., Byun J.Y., Mun H., Shmin W.B., Li T., Kim M.G., Kim A. (2014). A regeneratable, label-free, localized surface plasmon resonance (LSPR) aptasensor for the detection of ochratoxin A. Biosens. Bioelectron..

[28] Schroeder H.W., Cavacini L. (2010). Structure and function of immunoglobulins. J. Allergy Clin. Immunol..

[29] Miller J.C., Miller J.N. (2005). Statistics and Chemometric for Analytical Chemistry.

[30] Camacho S.A., Sobral-Filho R.G., Aoki P.H.B., Constantino C.J.L., Brolo A.G. (2017). Immunoassay quantification using surface-enhanced fluorescence (SEF) tags. Analyst.

[31] Choi J., Gani A.W., Bechstein D.J.B., Lee J.R., Utz P.J., Wang S.X. (2016). Portable, one-step, and rapid GMR biosensor platform with smartphone interface. Biosens. Bioelectron..

[32] Huang C., Wen T., Shi F.J., Zeng X.Y., Jiao Y.J. (2020). Rapid detection of IgM antibodies against the SARS-CoV-2 virus via colloidal gold nanoparticle-based Lateral-flow assay. ACS Omega.

[33] Chinnadayyala S.R., Park J., Abbasi M.A., Cho S. (2019). Label-free electrochemical impedimetric immunosensor for sensitive T detection of IgM rheumatoid factor in human serum. Biosens. Bioelectron..

[34] Liu L.L., Marques L., Correia R., Morgan S.P., Lee S.W., Tighe P., Fairclough F., Korposh S. Human IgM detection using an optical fibre long period grating sensor. Proceedings of the 2017 IEEE Sensors.

[35] Zhang Z., Wang X., Wei X., Zheng S.W., Lenhart B.J., Xu P., Li J., Allbrecht H., Liu C. (2021). Multiplex quantitative detection of SARS-CoV-2 specific IgG and IgM antibodies based on DNA-assisted nanopore sensing. Biosens. Bioelectron..

[36] Ortega G.A., Perez-Rodríguez S., Reguera E. (2017). Magnetic paper-based ELISA for IgM-dengue detection. RSC Adv..

[37] Campos R.A., Monroy K.L.J., Dilien H., Cleij T.J., Grinsven B., Eersels K. (2021). Imprinted polymers as synthetic receptors in sensors for food safety. Biosensors.

