# Supplementary material for: Surface Plasmon Resonance-Based Immunosensor for Igm Detection with Gold Nanoparticles
Source: Micromachines (Basel). 2021 Sep 10;12(9):1092. doi: 10.3390/mi12091092 (PMC8468630; doi:10.3390/mi12091092)
Supplement: Supplementary file 1 [file micromachines-12-01092-s001.zip › micromachines-1356258 - supplymeantry.pdf]

Supplementary Materials: Surface Plasmon Resonance-Based Immunosensor For IgM Detection With Gold Nanoparticles

Nilay Bereli [1], Monireh Bakhshpour [1], Aykut Arif Topcu [2] and Adil Denizli [1,*]

[1] Chemistry Department, Hacettepe University, Beytepe, 06800, Ankara, Turkey; bereli@hacettepe.edu.tr (N.B.); monir.b1985@gmail.com (M.B.)
[2] Medical Laboratory Program, Vocational Scholl of Health Service, Aksaray University, 68100, Aksaray, Turkey; aykuttopcu@aksaray.edu.tr
* Correspondence: denizli@hacettepe.edu.tr; Tel.: +90-31-297-7983; Fax: +90-312-299-2163

The effect of pH on IgM detection using the I-SPR sensor was examined in the range of pH 4.0-8.0 (Figure S1). The sensing surface was equilibrated with the desired pH buffer solutions after that 100 ng/mL IgM solution was interacted with I-SPR sensor.

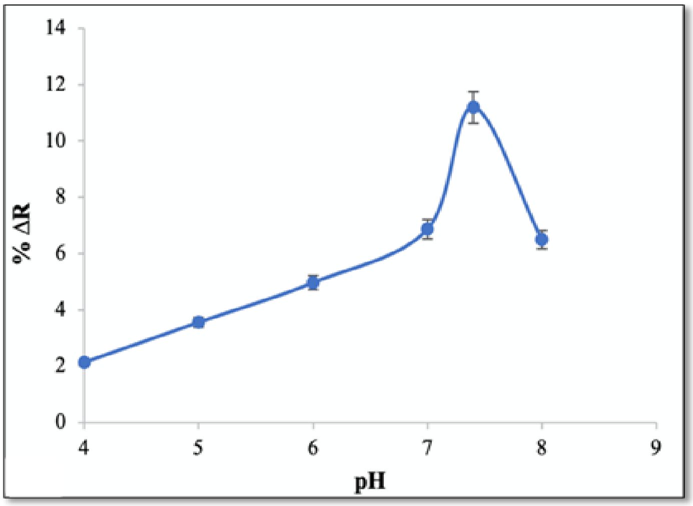

Figure S1. The effects of pH on the detection of IgM using I-SPR sensor (flow rate: 150 μL/min, 100 ng/mL IgM solution and T: 25 ºC).

Adsorption isotherm models can be used to understand the adsorption behavior during the adsorption process, thereby, the Langmuir, the Freundlich and the Langmuir-Freundlich adsorption isotherm models were investigated to understand the binding event manners of IgM. The Langmuir adsorption model is a homogenous adsorption model and defines the occurrence of adsorption on a monolayer surface with equal binding sites, whereas the Freundlich adsorption model defines a heterogenous adsorption on a heterogeneous surface. The Langmuir-Freundlich adsorption model is generally used to understand the adsorption behavior of molecular imprinting studies and is a useful model over the high concentrations. Hence, we used 3 adsorption isotherm models and the Langmuir, the Freundlich and the Langmuir-Freundlich models were calculated with Eqs. (1), (2), and (3), respectively.

$$\Delta R = [\Delta R_{max} (C) / K_D + (C)] \qquad \text{Eq (1)}$$

$$\Delta R = [\Delta R_{max} (C)^{1/n}] \qquad \text{Eq (2)}$$

$$\Delta R = [\Delta R_{max} (C)^{1/n} / K_D + (C)^{1/n}] \qquad \text{Eq (3)}$$

Herein, C; IgM concentration (ng/mL), ΔR_{max}; the sensor response, K_D; equilibrium dissociation constant and 1/n; the Freundlich exponent.

According to experimental results (Table S1), the binding manners of IgM and I-SPR obeyed the Langmuir adsorption model with a monolayer surface and equal binding sites with a high R^2 (0.99) value.

Langmuir model with ΔRmax equals 12.5 is found to be the most appropriate modal to identify the interaction between IgM molecules and I-SPR sensor.

Table S1. The results of the Langmuir, the Freundlich and the Langmuir-Freundlich adsorption isotherm models.

Langmuir	Freundlich	Langmuir- Freundlich
ΔR_{max} 12.5	ΔR_{max} 4.15	ΔR_{max} 19.26
K_A, ng/mL 0.033	1/n 0.54	K_A, ng/mL 0.028
K_D, mL/ng 30.01	R^2 0.85	K_D, mL/ng 35.37
R^2 0.99		R^2 0.94
